# An algorithm strategy for precise patient monitoring in a connected healthcare enterprise

**DOI:** 10.1038/s41746-019-0107-z

**Published:** 2019-04-30

**Authors:** Xiao Hu

**Affiliations:** Department of Physiological Nursing, Department of Neurological Surgery, Bakar Computational Health Sciences Institue, UCB-UCSF Joint Bioengieering Graduate Program, University of California, San Francisco, San Francisco, CA 94122 USA

**Keywords:** Translational research, Data mining

## Abstract

This perspective paper describes the building elements for realizing a precise patient monitoring algorithm to fundamentally address the alarm fatigue problem. Alarm fatigue is well recognized but no solution has been widely successful. Physiologic patient monitors are responsible for the lion’s share of alarms at the bedside, most of which are either false or non-actionable. Algorithms on patient monitors lack precision because they fail to leverage multivariate relationship among variables monitored, to integrate rich patient clinical information from electronic health record system, and to utilize temporal patterns in data streams. Therefore, a solution to patient monitor alarm fatigue is to open the black-box of patient monitors to integrate physiologic data with clinical data from EHR under a four-element algorithm strategy to be described in this paper. This strategy will be presented in this paper in the context of its current status as described in our prior publications.

## Introduction

In late year 2012 amid the height of medical device alarm fatigue crisis, my colleague Barbara Drew designed and conducted an observational study that collected all patient monitor alarms from 77 adult intensive care unit (ICU) beds of UCSF Medical Center in a period of one month. Furthermore, she and her team of highly trained PhD students with acute care nursing practice annotated approximately 12,000 ECG arrhythmia alarms of six most important kinds by reviewing physiologic signals that triggered these alarms. In total, more than 2.5 million alarms were recorded and the accuracy of the six annotated types of arrhythmia alarms was between 3.3% and 68.7% with ventricular bradycardia alarm being the least accurate and ventricular fibrillation the most accurate.^1^ These findings are on par with results from other studies in terms of alarm frequency but also clearly documented the state of the art of the current physiologic patient monitor algorithms as being excessive in number of poor quality alarms they generate.

Among the six types of annotated alarms, ventricular fibrillation and asystole alarms, when they are true, undoubtedly demand immediate attentions of clinicians. Appropriate clinical actions are less clear for the other four kinds of ECG arrhythmia alarms including ventricular bradycardia, pause, ventricular tachycardia, and accelerated ventricular rhythm. Therefore, researchers in the UCSF ECG Monitoring Lab now led by Michele Pelter conducted chart reviews to ascertain if and what actions were taken following those annotated true alarms of accelerated ventricular rhythm. No traces of relevant responses to these true alarms could be found in patient charts and hence these results imply that these alarms could have been of unclear values to clinicians. While our group has not systematically studied the impact of an excessive number of alarms from patient monitors and other medical devices on patients, anecdotes of confusions, stresses, disruption of sleep are abundant.

**Fig. 1:**
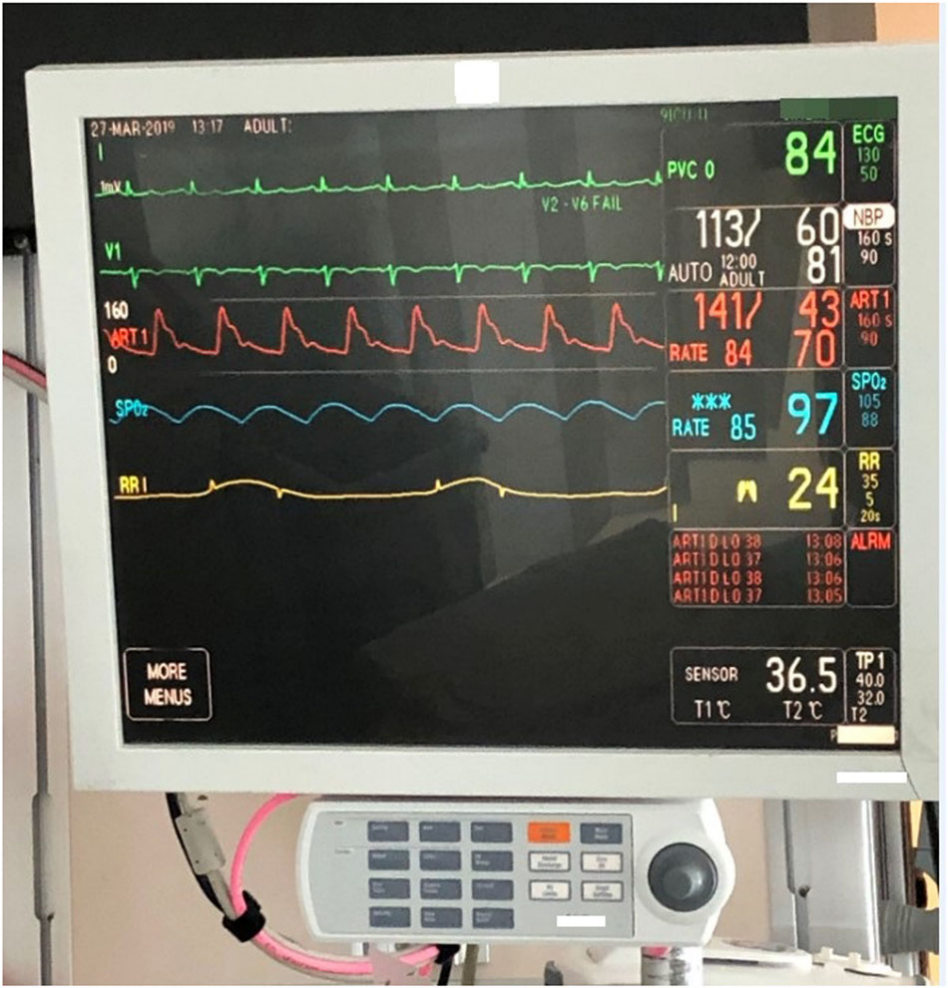
A photo of a bedside patient monitor in use at a medical surgical intensive care unit of the UCSF Medical Center. The salient features of the display include: (1) 6 s of multiple channel physiologic signals; (2) vital signs and their upper and lower threshold for alarming; (3) text of last four alarms. In addition, a panel of multiple buttons to access all monitor features and configurations is attached. The look-and-feel of this display has largely remained unchanged for decades as well as core ECG signal processing and arrhythmia detection algorithms

A large number of poor quality alarms derail the intended goal for patient monitoring as a first line of defense to recognize patient state changes. According to a recent systematic review of patient monitor alarm fatigue and the interventions to address the problem,^2^ reduction of the number of alarms instead of improving alarm quality has been the primary target—therefore the intervention often tweaked an existing system without fundamentally transforming the core of patient monitoring technology—the embedded signal processing algorithms that detect various conditions for alarming. Figure 1 shows a picture of a bedside patient monitor that is currently used in ICUs of our medical center. The look and feel, as well as core algorithms of these patient monitors, have gone at best evolutional changes. It is no surprise that alarms from patient monitors are still of poor quality.

Given the rapid adoption of electronic health record (EHR) systems and advancement of data science, the opportunities and potentially transforming success are within the reach to undertake the challenge of improving core patient monitoring algorithms by taking advantage of an ever increasingly connected technological ecosystem in a healthcare enterprise. By pursuing fundamental algorithm research, not only the problem of alarm fatigue will be solved but also a more predictive monitoring of patient state will be achieved to enable proactive interventions.

Our group has conducted several studies towards making patient monitor alarms more predictive of impending cardiopulmonary arrests by analyzing alarm data, physiologic signals, and the integration with EHR data. However, these studies were largely conducted in an opportunistic way whenever a new algorithm idea emerged. In particular, our first study^3^ introduced a concept that was termed SuperAlarm whose original definition referred to patterns of co-occurrences of individual alarms that are predictive of impending cardiopulmonary arrests and at the same time are infrequently found among control patients. SuperAlarm became the overarching brand name of our subsequent studies and its scope has been expanded beyond analyzing just alarms as will be discussed in this perspective. Realizing that innovating core algorithms for patient monitoring is challenging and can be better aided by a roadmap, we hence synthesize our previous work into a four-element algorithm strategy under which future innovations can be conceived and pursued in a more systematical way towards achieving precise patient monitoring.

## Methods

### Define precise patient monitoring requriement

To motivate our proposed algorithm strategy, we would like to first define three requirements for the future state of patient monitoring: (1) *Precision*. By precision, alarms from future patient monitoring solutions are required to be true with a high probability. An additional implication for being precise requires these alarms to be actionable. It is highly desirable for future alarms to be specific to certain patient conditions—e.g., sepsis alarms. But this is not a must-have because these alarms just need to succeed in drawing clinicians’ attention to patients at the right time for further diagnostic work-ups. (2) *Predictive*. By being predictive, it is not satisfying only for future alarms to detect patient crisis when it is happening, e.g., ventricular fibrillation alarms would be considered too late and future alarms need to be predictive of impending patient crisis. (3) Interpretable. By being interpretable, clinical and physiologic data that are responsible for the alarming condition should be traceable to support further diagnostic work-up.

### Four algorithm elements

To meet the above three requirements for future precise patient monitoring solutions, a paradigm shift from running algorithms only within the physical box of patient monitors by the bedside should be promoted so that the algorithms will be hosted in a connected, adequately resourced, and secure environment. The technical capacity to build such an environment does exist and hence we envision that the implementation of the proposed algorithm elements can take advantage of this technical reality.

The first algorithm element is to suppress as many as possible false alarms from the existing patient monitors. It is expected that existing bedside patient monitors will continue to exist for a foreseeable future. To maximize the use of these patient monitors in developing precise patient monitoring solutions, it will be rewarding to interrogate the physiologic signals that are time-aligned with alarms to determine whether a particular alarm should be suppressed. This approach is possible because one major reason for false alarms is that these alarms are often determined by analyzing one physiologic signal stream without corroborating with information that could be derived from other relevant physiologic signal streams. A stereotypical example is having false ventricular fibrillation alarms with regular hemodynamic pulses. There have been a large body of existing work dedicated to this algorithm strategy.^4–6^ Because missing true critically important alarms is unacceptable, the requirement for these algorithms is to avoid missing any true alarms while suppressing as many as possible false ones. However, because false alarm recognition would be accomplished after they are already annunciated by monitors, we are not truly suppressing false alarms and subsequent processing of alarms that are considered true is critical for the intended goal of improving precision of these alarms. This topic has not been well discussed in literature. However, using the proposed algorithm strategy, false alarm recognition can be readily integrated with other algorithm elements towards achieving precise patient monitoring.

The second algorithm element may seem paradoxical as it calls for designing new “alarms” toward precise patient monitoring. Even though the current physiologic patient monitors can generate thousands of alarms per bed per day, these alarms miss many predictive patterns that could have been derived from a deeper analysis of the physiologic data streams including ECG and other hemodynamic signals. In our published studies, we revealed that trending patterns in several ECG metrics including PR interval can be monitored to generate alarms that detect monotonic trends hours ahead of impending cardiopulmonary arrests.^7^ In our earlier work related to intracranial pressure (ICP), we showed the potential of analyzing ICP pulse morphology to detect signatures of impending ICP elevation minutes ahead.^8^ Because we are envisioning that our precise patient monitoring algorithm will be hosted in a connected environment, therefore, one should not be limited by only having access to physiologic data. Instead, these new “alarms” can be derived from additional sources, in particular, the EHR. As an early example, we were able to integrate tokenized abnormal lab test results from EHR with patient monitor alarms to achieve a better algorithm of predicting patient deterioration.^9^ Following the same strategy, other data modalities in EHRs can be tokenized as discrete alarms. For example, to tokenize surgical procedures, one could combine the type of a procedure and the beginning time of the procedure into one token. As another example, natural language processing can be applied to clinical notes to derive thematic topics and attach them with appropriate timestamps. This simple tokenization algorithm for EHR data is straightforward but powerful to represent any EHR data modality.

Through the pursuit of the first two algorithm elements, we leverage existing patient monitoring technologies to the maximal extent by suppressing false alarms from them and extracting additional alarms from analyzing the physiologic signals. Furthermore, a potentially rich set of alarms, which are obtainable through a tokenizing process, from EHR is available. Then the third algorithm element is to combine these individual alarms. Towards this end, one example algorithm is called SuperAlarm. This algorithm identifies alarm co-occurrence patterns^3^ that satisfy two criteria: (1) these patterns occur frequently before a clinical event of interest; (2) these patterns occur much less frequently among control patients. These SuperAlarm patterns can be identified by first using frequent itemset algorithm^10^ to identify candidates that frequently precede a target clinical event and then a filtering process to remove candidates if they frequently occur among control patients. Each SuperAlarm pattern will contain >1 individual alarms that co-occur in a time window—the characteristic of this window will be learned through training. These SuperAlarm patterns, therefore, capture high-order interactions among the alarms and enjoy the following desirable features: (1) SuperAlarm patterns further suppress false or nuisance alarms by cross-checking multiple data modalities. For example, we frequently noted that SuperAlarm patterns consist of ECG arrhythmia alarms and alarms related to hemodynamic status. These patterns have face validity because ECG arrhythmia with hemodynamic consequence is likely to be true and more clinically meaningful; (2) SuperAlarm patterns capture trending patterns. For example, pulse oxygenation level is typically calculated by proprietary algorithm and the raw values of these calculations are not available at a high frequency to enable trend detection. However, a SuperAlarm pattern consisting of SPO2 alarms that are triggered at different thresholds is likely related to a trending pattern of pulse oxygenation; (3) SuperAlarm patterns are multivariate by design, therefore, they characterize relationship between clinical data and physiologic data without having to rely on prior knowledge; (4) SuperAlarm patterns are understandable by clinicians and can support further diagnostic work-ups. It should be pointed out that frequent itemset algorithms do not count for the order of alarms to compose SuperAlarm patterns. This is a desirable feature because timing of EHR data is often determined by clinical logistics, therefore, imposing strict temporal order may lead to false discoveries not generalizable.

Once a set of SuperAlarm patterns are identified, these patterns can be deployed to monitor data streams from patient monitors and EHR system so that the arrival of a new data sample triggers a pattern-matching process to detect if any SuperAlarm patterns emerge. If a SuperAlarm pattern is detected, a corresponding SuperAlarm trigger will be generated. Continuing this process generates a sequence of SuperAlarm triggers. However, it is not optimal to use individual SuperAlarm trigger to alert clinicians. SuperAlarm patterns can be redundant, for example, a pattern consisting of heart rate >120 bpm and mean arterial blood pressure (ABP) >110 mmHg is a synonym to a SupeAlarm pattern consisting of tachycardia and mean ABP >110 mmHg. Furthermore, a patient condition of interest may progress at a pace that outruns the temporal scale of individual SuperAlarm patterns. For example, some etiologies behind cardiopulmonary arrest may take hours to develop and hence its prediction needs to accumulate information from time points that lag the current time. The fourth algorithm element, therefore, is to develop pattern recognition approaches to process sequences of SuperAlarm triggers. To analyze a sequence of SuperAlarm triggers, one draws an analogy between a sequence of SuperAlarm riggers with a text document where each SuperAlarm pattern could be considered as a unique word. Therefore, the analysis of sequences of SuperAlarm triggers could leverage all the classical as well as more recently developed statistical text analysis approaches. Because these sequence analysis approaches can be also applied to sequence of raw alarms, one could question the need of identifying SuperAlarm patterns. By using the same sequence representation and machine learning model to analyze sequence of raw patient monitor alarms and sequence of SuperAlarm triggers, our study showed that sequences of SuperAlarm triggers were much more predictive of patient deterioration towards cardiopulmonary arrest.^11^ Hence this study was able to support the notion that identification of SuperAlarm patterns is a beneficial step prior to sequence pattern recognition.

### Integration of the algorithm elements

**Fig. 2:**
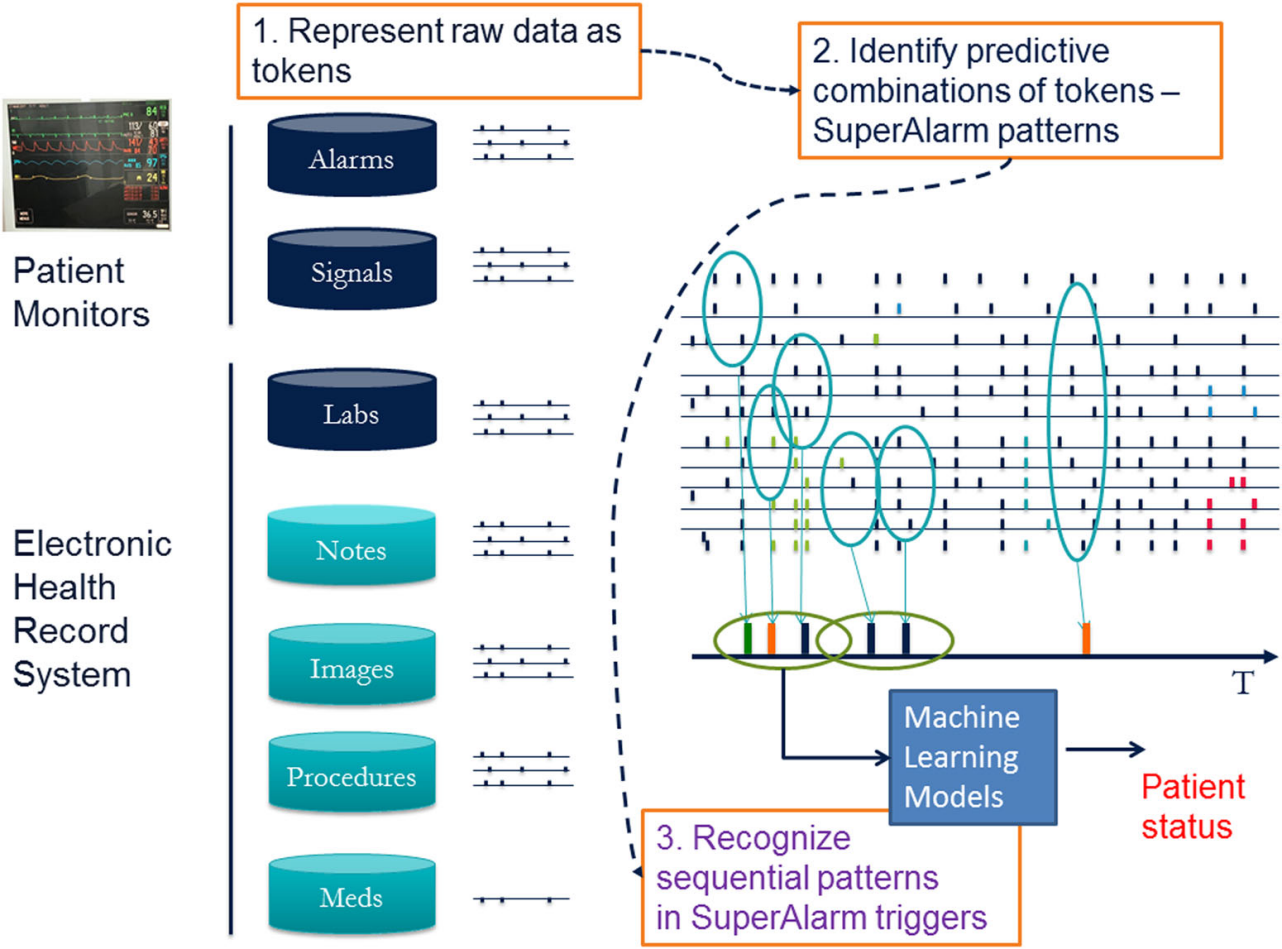
A schematic representation of integrating data from patient monitors and electronic health record (EHR) system toward a precise patient monitoring solution. The integration takes three key steps. The first step involves representing raw data as time-stamped tokens, the second step then uses training data to identify predictive patterns of co-occurring tokens, and the last step monitors and detects the emerging patterns from the data streams in real-time and further processes the sequence of these pattern triggers to characterize the patient status

Figure 2 illustrates an integration of the four algorithm elements into a precise patient monitoring solution. A key unifying step is the representation of all potential input data as “alarms”. Under this common representation, algorithm elements 3 and 4 can be invoked to identify predictive patterns across different data modalities as SuperAlarm patterns and then across the temporal dimension using sequence pattern recognition approaches.

### Case and control data for developing precise patient monitoring solutions

The algorithm solution as presented in Fig. 1 consists of several learning components. For example, there is a need to learn specific tokenization algorithms for different data modalities. In addition, SuperAlarm patterns and proper representation of sequences of SuperAlarm triggers are not a given *priori* and hence need to be learned. Finally, the sequence pattern recognition problem is a well-defined machine learning problem. Supervised learning approaches are particularly suited for these learning problems. Hence, a case-control learning data set will be needed. The case data should be from patients who experienced a clinical event. Because the primary objective of the patient monitoring is not to provide precise diagnosis without clinicians in the loop, the target clinical event can be a broad event, e.g., a common end point with heterogeneous etiologies. The control data should be from case patients that represent a baseline state—typically from a time zone that is further away from the time of target clinical events or from patients who never experience the target events but with matching clinical conditions. Because treatments often alter patient trajectory that would have led to the target events, it is infeasible to guarantee that control data thus collected will always correspond to a “healthy” state. We, therefore, propose that prospective validation and refinement should be an essential phase following the initial offline training. In the prospective validation phase, clinicians will be in the loop to help adjudicate detections or misses from running the solution. By incorporating just-in-time knowledge of clinicians with patients in front of them, the adjudication of the output from the algorithm will be more representative of the real-world expectation than only adjudicating the detections based on whether the target events occur or not.

## Discussion

We systematically synthesize our prior work in developing various algorithms for patient monitoring under a four-element strategy towards fundamentally solving patient monitor alarm fatigue problem and achieving precise patient monitoring. One key vision for this proposed solution is that future patient monitoring algorithms should not be confined to the physical box of bedside patient monitors. The requirements for precise patient monitoring solution will be better satisfied by integrating a rich set of clinical data from EHR with data from physiologic patient monitors. The capacity to stream data from bedside patient monitors and EHRs is becoming more readily available to enable this integrated patient monitoring. With the fast development of machine learning and deep learning techniques, development of precise patient monitoring solutions can benefit from having a common data representation for all relevant data from different sources. There are other possible ways of integrating the types of data that are discussed here.^12–14^ However, several advantages of our alarm-based representation should be highlighted. First, the co-occurring patterns are human readable and hence could facilitate further decision support once clinicians are notified of patients with concerning SuperAlarm patterns. Second, our proposed framework is modular and can be customized according to the specific local environment to run the system and to the specific problems at hand. Third, the proposed framework can take procedures and medications that patients receive as input. This is a tremendous advantage to use not only observations of patients but also the actions to the patient to infer patient state.

One area that our proposed framework has not covered is the potential to further personalize the monitoring algorithms through online learning with clinicians in the loop as they adjudicate the detections for a given patient. Because patients under acute care may need monitoring for an extended period of time, incorporating an online learning element could prove to be worthwhile. In closing this perspective, a speculated roadmap is presented in Table 1 for key technical and clinical characteristics in the future patient monitoring systems. To reach the desired future state of precise patient monitoring practice, we believe fundamental algorithm research need to drive the development and be led by a highly collaborative interdisciplinary team with both clinical and engineering expertise.

**Table 1:** List of key technical and clinical characteristics of the existing patient monitoring solutions in contrast with those speculated to exist in futuristic patient monitoring solutions in a 5/10 years horizon

Key technical and clinical characteristics of patient monitoring solutions	Past	Now	5 years+	10 years +
How are sensors attached to patients and connected to monitors?	Wired	Mostly wired	Wireless and wearable	Wireless and wearable
Do monitors support outbound data streaming?	Not supported	Supported in proprietary format	Supported in standard format	Supported in standard format
Where is streaming data analysis being done?	Within monitors	Within monitors	Within monitors+on premise servers	Within monitors+on premise servers+in cloud
Is monitoring algorithm predictive of clinical events with appropriate lead time?	No	No	Yes	Yes
Can monitoring algorithm learn in an online fashion?	No	No	Yes	Yes
Does monitoring algorithm provide diagnostic decision support?	No	No	Only partially	Yes
Does monitoring algorithm provide therapeutic decision support?	No	No	No	Yes
